# Inter-Individual Difference in the Effect of Mirror Reflection-Induced Visual Feedback on Phantom Limb Awareness in Forearm Amputees

**DOI:** 10.1371/journal.pone.0069324

**Published:** 2013-07-25

**Authors:** Noritaka Kawashima, Tomoki Mita, Masahiro Yoshikawa

**Affiliations:** 1 Department of Rehabilitation for the Movement Functions, Research Institute, National Rehabilitation Center for Persons with Disability, Tokorozawa, Saitama, Japan; 2 Department of Prosthetics and Orthotics, Research Institute, National Rehabilitation Center for Persons with Disability, Tokorozawa, Saitama, Japan; 3 Intelligent Systems Institute, National Institute of Advanced Industrial Science and Technology (AIST), Tsukuba, Ibaragi, Japan; University of Salamanca- Institute for Neuroscience of Castille and Leon and Medical School, Spain

## Abstract

**Objective:**

To test whether the phantom limb awareness could be altered by observing mirror reflection-induced visual feedback (MVF) in unilateral forearm amputees.

**Methods:**

Ten unilateral forearm amputees were asked to perform bilateral (intact and phantom) synchronous wrist motions with and without MVF. During wrist motion, electromyographic activities in the extensor digitorum longus (EDL) and flexor carpi radialis muscles (FCR) were recorded with bipolar electrodes. Degree of wrist range of motion (ROM) was also recorded by electrogoniometry attached to the wrist joint of intact side. Subjects were asked to answer the degree of attainment of phantom limb motion using a visual analog scale (VAS: ranging from 0 (hard) to 10 (easy)).

**Results:**

VAS and ROM were significantly increased by utilizing MVF, and the extent of an enhancement of the VAS and wrist ROM was positively correlated (r = 0.72, p<0.05). Although FCR EMG activity also showed significant enhancement by MVF, this was not correlated with the changes of VAS and ROM. Interestingly, while we found negative correlation between EDL EMG activity and wrist ROM, MVF generally affected to be increasing both EDL EMG and ROM.

**Conclusions:**

Although there was larger extent of variability in the effect of MVF on phantom limb awareness, MVF has a potential to enhance phantom limb awareness, in case those who has a difficulty for the phantom limb motion. The present result suggests that the motor command to the missing limb can be re-activated by an appropriate therapeutic strategy such as mirror therapy.

## Introduction

A phantom limb is the sensation that an amputated limb is still attached to the body and is moving with other body parts [1], [2]. While some amputees have a vivid kinesthesia for their phantom limb, previous studies have described others as having an awareness of the missing limb as clenched and paralyzed in a specific position [3], [4]. One possible interpretation for the latter case is that the amputee cannot send motor commands to the missing limb. This interpretation can work under the premise that the patient does not have a motor representation of the missing limb anymore. However, given the previous findings that the awareness of the phantom limb can be enhanced with viewing an image of their intact hand, which can create a visual illusion of their missing hand (i.e. the “mirror box”) [3], [5], it is reasonable to consider that the patients still possess the ability to send motor commands to the missing part. With regard to this point, Mercier et al. [6] have previously reported an interesting result that the sense of phantom limb motion can be re-awakened through transcranial magnetic stimulation on the motor cortex, even in the case the patient has a “paralyzed” phantom limb.

The mirror box, invented by Dr. Ramachandran [3], is based on the concept that the visual feedback regarding the missing limb enhances an awareness of phantom limb. Previous studies reported that the mirror therapy can reduce phantom pain [7]–[11] and enhance an awareness of the phantom limb when the patients see the reflection of the intact hand [5]. Although the above mentioned findings provide evidence supporting an effectiveness of mirror therapy on phantom pain, the mechanisms underlying this is not fully described. Obviously, mirror reflection-induced visual feedback (MVF) itself affects phantom limb awareness and kinesthesia which might have relevance to the mechanism underlying pain relief. As far as we know, there is little number of studies which examine the changes of the phantom limb awareness and/or perception due to MVF.

The question we would like to ask here is how the MVF affects the phantom limb awareness and its intended motion. In order to evaluate the phantom limb motion, we recorded muscle electromyographic (EMG) activity of the residual wrist flexor and extensor muscles near the amputation stump. With regard to EMG measurements, Reilly et al. [4] have recently recorded the EMG activities of the residual muscles in the upper amputees and reported that different intention of the phantom limb movements are associated with distinct muscle EMG activity in the residual stump muscles. We also confirmed that the forearm stump muscles demonstrated clear EMG activities that correlated with the phase of phantom wrist motion [12]. We therefore assumed that changes in phantom limb motion can be quantitatively evaluated by EMG recordings. The purpose of this study was therefore to test whether phantom limb kinesthesia altered by observing MVF. Since previous literature indicated individual differences of the effect of MVF on phantom limb pain, we additionally paid attention to clarify this point. A better understanding regarding the theoretical basis for mirror therapy might enable us to determine who will have benefit of it and to prescribe protocol based on patient's characteristics.

## Methods

### Subjects

Thirteen unilateral forearm amputees participated in the present study (age: 56.5+–16.49 yrs). Ten subjects suffered work-related traumatic injury, two had car accidents (Subject C and K), and other one subject amputated as the result of bone cancer (Subject L). Subject D experienced a 5-day delay between his traumatic injury and the amputation. This was the only subject with a history of pre-amputation paralysis. Each subject was interviewed by one author (M.T.). The interviews documented medical history, present residual-limb (stump) sensations and pain, condition of phantom limb, phantom pain. Table 1 provides details of the subjects. Since all subjects had undergone unilateral forearm amputation, stump length (distal to proximal end of the radius) was included in Table 1. The experimental protocol of this study was approved by the research ethics boards of NRCD. Written informed consent was obtained from all subjects before participation in the study.

**Table 1:** Patients characteristics.

					Phantom limg						
Subject	Age	Amputation side	Dominant hand	Stump length	Awareness	pain	numbness	Stump pain	Telescoping	Cause of amputation	Daily prosthesis use
Patient A	65	Left	Right	190	+	–	+	–	+	trauma	cable operated
Patient B	33	Right	Left	150	+	–	+	–	+	trauma	cable operated & cosmetic
Patient C	76	Left	Right	110	+	–	+	–	+	trauma	cable operated
Patient D	64	Right	Right	95	+	–	+	+	+	trauma	cable operated & cosmetic
Patient E	65	Right	Right	112	+	+	+	–	–	trauma	myoelectric
Patient F	33	Right	Right	125	+	+	+	–	+	trauma	none
Patient G	43	Left	Right	150	+	+	+	+	+	trauma	cosmetic
Patient H	63	Right	Right	209	+	–	+	–	+	trauma	cable operated
Patient I	77	Right	Right	88	+	–	–	–	+	trauma	cable operated
Patient J	71	Right	Right	140	+	–	–	–	+	trauma	cable operated
Patient K	63	Left	Right	125	+	–	–	–	+	trauma	cosmetic
Patient L	51	Left	Right	88	+	–	–	–	+	trauma	myoelectric & cosmetic
Patient M	31	Right	Right	135	+	+	+	–	+	trauma	myoelectric & cosmetic

### Mirror reflection-induced visual feedback (MVF)

**Figure 1:**
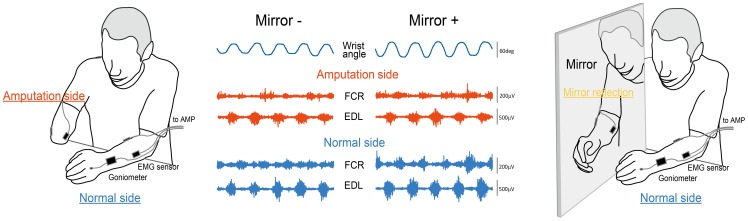
Experimental setup. Subject was asked to conduct bilateral wrist motion with (right) and without (left) visual feedback of the phantom limb by way of a mirror reflection of the intact hand. Middle panel shows waveforms of the wrist joint motion and EMG activity of the FCR and EDL muscles during rhythmic synchronous wrist flexion-extension movement.

MVF utilizes a simple contraption in which a mirror provides reflection-induced visual feedback of the phantom limb, which is described by Ramachandran and colleagues [13]. Subject placed his intact arm on one side of the mirror, which was positioned in such a way that he could see the reflection of the intact hand as another side of the hand (Figure 1). Subjects were instructed to perform synchronous and periodic (flexion to extension and vice versa) wrist motions using both intact and phantom limbs with the visual feedback.

### Measurements

In order to record the changes in phantom limb motion, we conducted an experiment which consisted of synchronous and periodic wrist motions using both intact and phantom limbs. During the experiment, subjects were asked to describe the difficulty of phantom limb motion using a visual analog scale (VAS: ranging from 0 (hard) to 10 (easy)). The subject was instructed to conduct the motion for 30 seconds at a comfortable speed. During the movement, bilateral muscle electromyographic (EMG) activity was obtained from the extensor digitorum longus (EDL) and flexor carpi radialis muscles (FCR) with bipolar electrodes. All subjects had EDL and FCR muscles. The EMG signal was amplified and band-pass filtered between 20 and 450 Hz (The Bagnoli-8 EMG System, DELSYS, USA). In order to measure changes in wrist angle, an electrogoniometer (Goniometer System, Biometrics Ltd., Ladysmith, VA, USA) was attached to the wrist joint on the intact side. Because we instructed subjects to move both wrists synchronously, we assumed that the motion of the phantom limb could be characterized by measuring the intact side motion. Since the EMG data were obtained on different days, we should be careful in comparing these data. We used bipolar electrodes (DE-2.3, DelSys, Inc., Boston, MA, USA) with a constant interelectrode distance (1 cm apart), and tried to place the electrodes at the same locations among the three experiments. Also, in order to reduce the impedance between electrode and muscle, skin preparation (abrasion, cleaning with alcohol) was carried out carefully before recording. During the experiment, all data were continuously monitored by Power Lab software (Chart ver. 5, AD Instruments, USA) and were digitized at 1 kHz for later analysis.

### Statistics

All values are given as the mean +− SD. Paired t-test was used to compare the above mentioned parameters between with (Mirror+) and without (Mirror−) MVF conditions. Cohen's d was also calculated to ensure the effect size of the sample. Correlation coefficient was calculated to test relationship among variables. Significance was accepted at p<0.05.

## Results

All subjects were able to move the wrist of the phantom limb during experimental session. Figure 1 shows a typical example of the waveform of the wrist joint angle recorded from the intact side and the EMG activity of the FCR and EDL muscles in both arms obtained from one patient.

### Phantom limb movement

**Figure 2:**
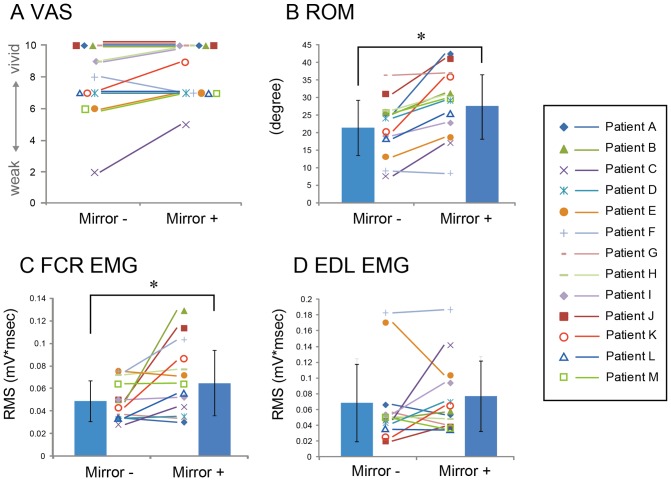
Effect of MVF on VAS (A), ROM (B), EMG activity of FCR (C) and ECR muscles (D). Each symbol indicates an individual subject data. The error bars indicate the standard deviation of the mean value. * Significant difference (p<0.05).

An awareness of the phantom limb motion recorded by VAS tended to be increased, but VAS showed ceiling effect for those who could move phantom limb without any difficulty. For patients answered highest (10) score irrespective to the use of MVF. As shown in the Figure 2B, the range of wrist joint motion was significantly larger in case the subject use MVF (Mirror– vs. Mirror+: 21.2±7.90 vs. 27.2±9.14 degrees, p<0.05, d = 0.99).

### EMG activity

As shown in Figure 2C, most of subject showed changes of the muscle activity between with and without MVF. For the FCR muscle, there is statistically significant differences between Mirror+ and Mirror– condition (Mirror– vs. Mirror+: 48.3±17.97 vs. 64.11±29.22, p<0.05, d = 0.92). For the EDL muscle, no consistent changes were observed between two conditions (Mirror– vs. Mirror+: 67.43±59.40 vs. 76.14±45.15, n.s., d = 0.26).

### Correlation among VAS, wrist ROM and EMG activities

**Figure 3:**
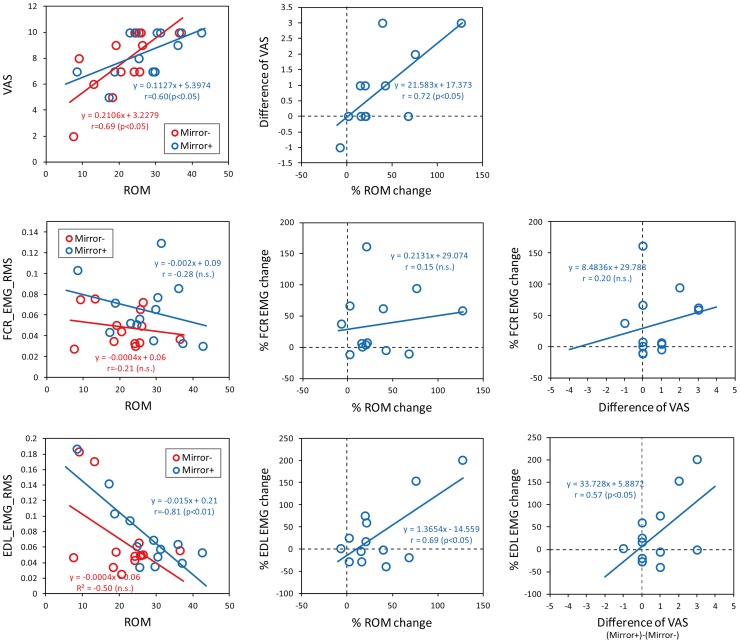
Summary of correlation analysis among VAS, wrist range of motion and EMG recorded from FCR and EDL muscles. In the left panel, blue and red circles indicate the absolute value of each parameter in Mirror– and Mirror+ conditions, respectively. In the center and right panels show the results of correlation analysis with the use of change rate induced by MVF. Regression line, the value of correlation coefficient and its statistical significance were indicated in each figure.

Figure 3 shows the results of correlation analysis among VAS, wrist range of motion and EMG recorded from FCR and EDL muscles. In the left panel, blue and red circles indicate the absolute value of each parameter in Mirror– and Mirror+ conditions, respectively. We found statistically significant positive correlation between VAS and ROM in both conditions (Mirror+: r = 0.60, p<0.05, Mirror–: r = 0.69, p<0.01). While FCR muscle did not show any relevance to wrist ROM, negative correlation was found between EDL EMG activity and wrist ROM in Mirror+ condition (r = 0.81, p<0.01).

In the center and right panels show the results of correlation analysis with the use of change rate induced by MVF. The results show positive correlation between MVF-induced changes of VAS and wrist motion (r = 0.72, p<0.01). Regarding the EMG activity, while MVF-induced change of FCR EMG did not have relevance to ROM and VAS, EDL EMG showed positive correlation with ROM (r = 0.69, p<0.05) and wrist ROM (r = 0.57, p<0.05), respectively.

## Discussion

The purpose of this study was to test whether the phantom limb awareness could be altered by observing mirror reflection-induced visual feedback (MVF) in unilateral forearm amputees. As shown in the Figure 2, an attainment of the phantom limb movement determined by VAS tended to be higher in case patients utilized MVF than they did not. However, it is obvious that VAS has ceiling effect in those who could move phantom limb even if they do not utilize MVF (4 of 13 patients answered highest score irrespective to the use of MVF). This result implies a limitation of VAS as a reliable probe to determine the effect of MVF on phantom limb awareness. A traditional and conventional way to characterize phantom limb is describing the patient's feeling as a case report and case series [7], [10], [13]. Except for brain imaging study, almost all outcome measures employed in the behavioral and clinical studies regarding the extent of motion and pain of phantom limb were subjective or psychological scale, such as visual analog scale. We here attempt to quantify the effect of MVF on the extent of phantom limb motion by utilizing the recording of wrist joint angle of the intact hand and EMG in the stump muscles while forearm amputees simultaneously move phantom and intact wrist. We confirmed that all subject showed clear EMG activity in the stump muscles in accordance with the phase of phantom wrist movement.

In the present study, while there was larger extent of inter-individual variability, all subjects possessed clear awareness of their phantom limb, and were able to move the wrist of phantom. As shown in the figure 1, EMG activities recorded from the FCR and EDL muscles shows clear modulation pattern in accordance with phantom limb motion. In the present study, most of patients who felt that MVF make them easy to move phantom limb had some extent of difficulty in their phantom limb movement in case they did not utilize MVF. This was shown as not only relatively lower score of VAS and smaller degree of wrist ROM, but negative correlation between EDL EMG activity and wrist ROM. This result might reflect that EDL muscle activity is not simply reflects the extent of phantom limb motion, but an effort to make motor command to phantom limb. Regarding the relationship between EMG activity and phantom limb motion, the present result showed that MVF generally affect to be increasing FCR EMG activity, but this was not correlated with the MVF-induced changes of VAS and ROM (Figure 3). On the other hand, MVF-induced change of EDL EMG positively correlated with that of ROM and VAS. These results imply one hand magnitude of EMG itself does not simply explain an attainment of phantom limb motion among subject, on the other hand, at least suggest that activation level of EDL muscle link to the enhancement of phantom limb motion.

With regard to EMG measurements, Reilly et al. [4] have recorded the EMG activities of the residual muscles in the upper amputees and reported that different intention of the phantom limb movements are associated with distinct muscle EMG activity in the residual stump muscles. We therefore assumed that changes in phantom limb motion can be quantitatively evaluated by EMG recordings. Previous studies have described the cases those who have an awareness of the missing limb as clenched and paralyzed in a specific position [4], [5], [13]. One possible interpretation for such cases is that the amputee cannot send motor commands to the missing limb. This interpretation can work under the premise that the patient does not have a motor representation of the phantom limb anymore. However, given the previous findings that the awareness of the phantom limb can be enhanced with viewing an image of their intact hand, which can create a visual illusion of their missing hand (i.e. the “mirror box”) [5], [13], it is reasonable to consider that the patients still possess the ability to send motor commands to the missing part. With regard to this point, Mercier et al [6] have previously reported an interesting result that the sense of motion in the phantom limb can be re-awakened through transcranial magnetic stimulation of the motor cortex, even when the patient has a “paralyzed” phantom limb. This finding strongly suggested that amputees preserve actual motor command to the missing parts. Considering these facts, it is likely that the visual feedback could be trigger to re-activate the motor command to the phantom limb.

**Figure 4:**
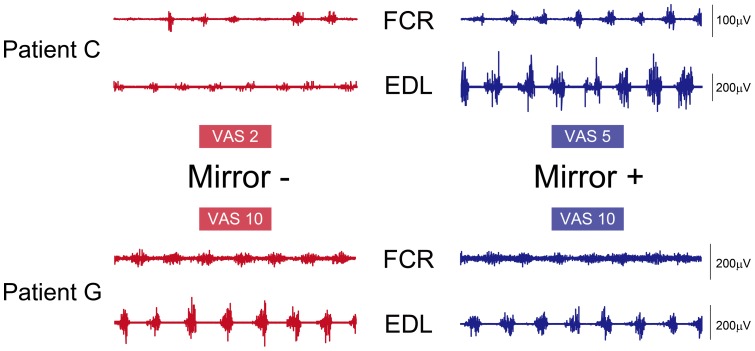
Representation of the different two types of EMG changes due to MVF. Patient C (top panel) showed remarkable increase of VAS and EMG amplitude due to MVF while Patient G (bottom panel) did not show obvious changes in both VAS and EMG activities.

Patient C in the Figure is a typical case who showed remarkable enhancement of phantom limb awareness with MVF. While he could not move well his phantom limb without MVF, profound increase of VAS, ROM, and EMG was observed when he utilized MVF (see top panel of figure 4). It is, at least in this case, reasonable to consider that the motor command to the missing limb and resultant evoked-sensory input can be enhanced by utilizing mirror reflection-induced visual feedback. Contrary to these cases, two patients reported that MVF make them to move their phantom limb difficult. Both patients could move their phantom limb as they intended. In fact, when they attempted to move the wrist of phantom limb while watching the mirror, but after several minute of trial, they tended not to see mirror. This may be attributed to the discrepancy between actual perception of the phantom limb and visual information [14]. In other words, MVF did not much make sense for them to enhance the phantom limb movement. The present results therefore suggest that MVF has a potential to enhance motor command to phantom limb and its awareness, in case those who has a difficulty for the phantom limb movement. This interpretation is good agreement with the previous description that “mirror therapy is particularly suited to those who find motor imagery difficult [15].

While we paid much attention to the awareness of the phantom limb, it is quite important to discuss for phantom limb pain based on our results. In this study, 3 of 10 patients possessed vivid phantom limb pain. We asked these patients to tell us VAS of phantom limb pain, and one patient (Patient G) reported the reduction of the pain. On the other hand, in case of Patient F, extent of phantom limb pain (numbness) increased by MVF which accompanied with decreased ROM. Further detailed analysis, specifically for EMG, should be needed to examine factors affecting phantom limb and pain and those interaction.

In conclusion, the present result suggested that (1) MVF has a potential to enhance phantom limb awareness, in case those who has a difficulty for the phantom limb motion, (2) the motor command to the missing limb might have a potential to be re-activated by an appropriate therapeutic strategy such as mirror therapy, and (3) the measurement of ROM from intact hand and EMG in the stump muscle gave us useful information for the discussion of the mechanisms underlying phantom limb.
